# Editorial

**DOI:** 10.1155/S1463924684000122

**Published:** 1984

**Authors:** Harold Egan

**Affiliations:** Laboratory of the Government Chemist Cornwall House Stamford Street London SE1 9NQ UK


### Editorial

### Method validation: the harmonization of collaborative analytical studies

The first International Symposium on the Harmonization of Collaborative Analytical Studies was held in Helsinki from 20 to 21 August 1981 [1 and 2]. This was in fact the first occasion on which many of the international organizations which publish compendia of analytical methods developed by the process of collaborative analytical studies had met together to compare philosophies. The meeting urged the presidents of the three sponsoring IUPAC Divisions to give positive attention to the need to develop international guidelines, to harmonize definitions of the basic parameters concerned and the philosophy of applying these to the validation process and to provide a continuing forum for the exchange of information in these fields. If nothing else, the symposium aroused a critical awareness that many different international groups were working, largely independently, in a field of fundamental and sometimes far-reaching significance in food, health, environmental quality, commercial, industrial specification and control. At the same time it is appreciated that analysis is often only an index of biological or other function and, for this reason alone, it is important that, in areas where approximations and uncertainties abound, the reliability of the analytical aspects be properly understood and matched to their purpose. Finally, there is an economic factor in terms of time, money and professional resources which calls for a need to maximize the application of these in a world ever more demanding of their services.

The first symposium also gave stimulus for further reflection on the present position and the different functions which the interlaboratory study based on collaborative exercises is based. A main distinction can be drawn between the use of a study to determine the attributes of a method for acceptance purposes and for performance purposes. A study may also be used to compare the attributes of several methods, to compare the ability of different analysts or to establish a consensus value for a reference material. Consideration of these aspects pointed to the need, more than ever, for criteria for the acceptance or validation of methods, particularly those used by enforcement authorities with the implicit need to maintain a sometimes delicate balance between fraud or hazard and usefulness and value. The challenge has been taken up in particular by the Association of Official Analytical Chemists, which decided, as part of its centennial arrangements in 1984, to establish a definitive protocol for the validation of methods by the process of collaborative interlaboratory study. The final first draft of this document was published last year [3]. There is no illusion that this is a perfect framework for validation, any more than that it is clear (and perhaps always has been clear) that total harmonization is neither practicable nor indeed necessarily desirable. Two parts to the AOAC strategy, which have both developed at different paces, can clearly be distinguished: the basic experimental framework and the manner in which results obtained using the framework are interpreted. Both of these aspects will be discussed further at the second harmonization symposium, to be held in Washington, D.C. from 25 to 27 October 1984, and sponsored jointly by IUPAC, the AOAC and the US National Academy of Sciences.

The objectives of the second symposium will be to identify the design essential for the validation of the performance of analytical methods, to identify where possible the minimum criteria for an interlaboratory study for validation in the light of this, and to compare the various factors and criteria identified in the design, as recognized by the various international and other organizations experienced in this field, many of which will be represented at the symposium, with the view where possible of harmonization on an international basis.

John Taylor has identified a hierarchy of analytical methodology [4] in which the term 'protocol' is seen as a set of definitive, mandatory directions, to be followed without exception. At the same time, he has recognized that it is impossible to describe each and every step with equal—not to say complete—absence of ambiguity or scope for individual interpretation. The aim of a method description must be to minimize the variability which can arise from this cause and it is partly for this reason that the concept of ruggedness has been introduced into the AOAC protocol referred to above. This raises wider issues such as the validation of the analyst who performs the method, or the accreditation of the laboratory systems in which he is working. All of these enter into the real world of applied analysis and for this reason the organizers of the second symposium have introduced a workshop element into the programme. In this way, it is hoped to gain a fuller insight into some of the practical problems which have been encountered, with a 'how to do it' element included.

Effective harmonization means that one organization can judge whether the validation of a method by another organization can be accepted without further testing: this would be to the advantage of all concerned.

### References

1. Mesley, R. J., *Proceedings of the Analytical Division, Royal Society of Chemistry*, **18** (1981), 461.
2. Egan, H., and West, T. S., *Collaborative Analytical Studies in Chemical Analysis* (Pergamon Press: Oxford, 1982).
3. Horwitz *et al.*, *Journal of the Association of Official Analytical Chemists*, **66** (1983), 455.
4. Taylor, J. K., *Analytical Chemistrv*, **55** (1983), 600A.

**Harold Egan**

*Laboratory of the Government Chemist, Cornwall House, Stamford Street, London SE1 9NQ.*

